# Protective effects of low-intensity pulsed ultrasound on aluminum overload-induced cerebral damage through epigenetic regulation of brain-derived neurotrophic factor expression

**DOI:** 10.1042/BSR20181185

**Published:** 2019-01-22

**Authors:** Juan Li, Dong-dong Zhang, Chao-qing Wang, Miao Shi, Liang-liang Wang

**Affiliations:** 1 Department of Special Clinical, Liaocheng People’s Hospital, No. 67 Dongchang West Road, Liaocheng City, Shandong Province 252000, China; 2 Department of Ultrasound, Liaocheng People’s Hospital, No. 67 Dongchang West Road, Liaocheng City, Shandong Province 252000, China; 3 Department of Functional, Zun Hua Second Hospital, Tangshan City, Hebei 064200, China

**Keywords:** aluminum-induced cerebral damage, BDNF, HDAC4, HDAC6, low-intensity pulsed ultrasound

## Abstract

In consideration of its noninvasive administration and endogenous stimulation property, the enhancement of brain-derived neurotrophic factor (BDNF) via low-intensity pulsed ultrasound (LIPUS) could be a novel strategy for aluminum (Al) overload-induced cerebral damage. LIPUS was pre-treated 7 days before concomitantly given with aluminum chloride (AlCl_3_) daily for a period of 42 days. Morris water maze and elevated plus maze were performed to analyze spatial learning and memory. Western Blot and immunoprecipitation were used to detect BDNF and histone acetylation of histone H3 lysine 9 (H3K9) and histone H4 lysine 12 (H4K12) in the hippocampus. Assay of malondialdehyde (MDA), superoxide dismutase (SOD), glutathione (GSH), and glutathione peroxidase (GSH-Px) indicated the extent of oxidative damages. Aluminium exposure in rats can cause attenuated spatial learning and memory, followed by up-regulated histone deacetylase 6 (HDAC6) expression, down-regulated H3K9 and H4K12 acetylation at the PIII and PIV promoter of BDNF, all of which will eventually inhibit BDNF expression. LIPUS can recover reduced cognitive function by restoring histone acetylation and BDNF expression, accompanied with increased SOD, GSH, and GSH-Px activity. LIPUS treatment might alleviate aluminium exposure-induced cognitive decline by acetylation regulation of BDNF expression and reducing oxidative stress in the hippocampus.

## Introduction

As the richest element in the crust of earth, increased and accumulated concentration of aluminum (Al) can be observed in the human brain during normal aging [1]. Aluminium salts have been considered as an unavoidable environmental factor to enter human body by various routes, which can increase the cellular oxidative microenvironment by its potential pro-oxidant properties [2], induce various biochemical changes and neurofilament changes after prolonged aluminium exposure, and result in cognitive disorders, such as progressive neurodegeneration in the hippocampus and cerebral cortex [3–5]. Aluminium salts are considered as powerful neurotoxicant and have also been testified to cause cholinergic terminals degeneration in the cortical areas and hippocampus, which may accumulate in the cingulate bundle and thereby induce cognitive deficiency and dementia [6].

Compared with pharmacological techniques, localized and noninvasive brain stimulation techniques show a direct effect on neural circuits, which can be widely used in basic science, translational, and clinical practices. Among them, low-intensity pulsed ultrasound (LIPUS) shows the property of high spatial resolution and noninvasive processes, which make it more popular. It is worth noting that LIPUS can induce the regeneration of axon *in vivo* peripheral nerve injury trials. It is also reported that LIPUS can stimulate the intact rat brain circuitry and promote expression of brain-derived neurotrophic factor (BDNF), which can regulate the long-term memory [7–9], to protect aluminium salt-induced cortex injury in Alzheimer’s disease model. Considering its noninvasive administration and endogenous BDNF stimulation character, LIPUS may point to a new way for cognitive deficiency and dementia disorders treatment.

In this context, aluminum chloride (AlCl_3_)-exposure-induced cognitive impairment and oxidative stress rats model are constructed to testify the neuroprotective effect of LIPUS.

## Methods and materials

### Animal

Male Sprague–Dawley (SD) rats (160–170 g) of specified pathogen-free grade were purchased from Shanghai Laboratory Animal Center (Shanghai, China). All rats were maintained in accordance with the Guidelines of Care and Use of Laboratory Animals published by the China National Institute of Health. All of the experimental procedures were approved by the Animal Care and Use Committee of Liaocheng People’s Hospital. SD rats were classified into four groups (control, LIPUS, AlCl_3_, and LIPUS plus AlCl_3_). Rats in LIPUS group were exposed to LIPUS every day for 42 days. In the AlCl_3_ group, AlCl_3_ (100 mg/kg; oral gavage) was administrated every day for 42 days. Seven days prior to AlCl_3_ treatment, LIPUS was administered and repeated daily in LIPUS plus AlCl_3_ group.

LIPUS plus AlCl_3_ group rats were anesthetized (2% isoflurane in 2 l/min oxygen) in the prone position, and a heating pad was used to maintain the body temperature. After mounting the rat heads to a stereotaxic apparatus (Stoelting, Wood Dale, IL, U.S.A.), LIPUS stimulation was applied around the top of the cranium.

### LIPUS apparatus

A piezoelectric transducer (A392S; Panametrics, Waltham, MA, U.S.A.) was set as 1 Hz, 5% duty cycle, and 50 ms burst lengths, which was then fixed with a stereotaxic apparatus and focused the acoustic beam to the 3.0 mm posterior and 2.5 mm lateral region of the bregma. The spatial-peak temporal-average intensity (ISPTA) was measured with a radiation force balance (RFB, Precision Acoustics, Dorset, U.K.) and was set as 528 mW/cm^2^. In order to maximize the transmission of the LIPUS (three sonications, 5 min duration, and 5 min interval), ultrasound transmission gel (Pharmaceutical Innovations, Newark, NJ, U.S.A.) was mounted to the detected region.

### Behavioral assessment

A total of 40 SD rats were classified into four groups (control, LIPUS, AlCl_3_, and LIPUS plus AlCl_3_), and all these rats were used to evaluate the effects of LIPUS on behavioral outcomes in AlCl_3_-treated rats. A custom-made escape plastic stand (20 cm in diameter on the circular top) was put approximately 2 cm above the surface of the water. The rats were carefully released in the water to face the direction of swimming pool wall, then the rats were led to the escape plastic stand and stayed there for 20 s, while the maximum acquisition time was set as 90s. The time needed by the rats to arrive at the escape plastic stand on the 20th day after AlCl_3_ exposure was recorded and defined as acquisition latency (AL). After that, the same escape plastic stand was released about 2 cm below the water level. The times needed to reach the escape plastic stand on the 21st day and 42nd day after AlCl_3_ exposure was recorded and defined as retention latency (RL), which was measured 1 day after AL was recorded, and the relevant rats were released randomly at one end of the edges facing the swimming pool wall.

The elevated plus maze, which had two open arms (50 × 12 cm) and was intersected with two closure walls, was raised 66 cm above the floor level. Rats were released at one side of the open arm facing outside of the center part of the elevated plus maze. On the 20th day after the exposure of AlCl_3_, the time needed by the rats to move from one open arm to the closed arm was defined as transfer latency (TL). Similarly, such behavioral performances were measured on the 21st and 42nd day.

### Western blot

SD rats were classified into four groups (control, LIPUS, AlCl_3_, and LIPUS plus AlCl_3_), 4 h after the last LIPUS stimulation, six rats were killed and fresh hippocampus tissues were homogenized by T-Per extraction reagent (Pierce Biotechnology, Inc.). Lysates were centrifuged and supernatants were harvested and assayed with Protein Assay Reagent (Bio-Rad, CA, U.S.A.). A 40 μg protein was placed on 4–20% SDS-PAGE, transferred to Immun-Blot^®^ PVDF membranes (Bio-Rad, CA, U.S.A.), blocked for 1 h with tris-buffered saline tween-20 (TBST)-containing 5% (w/v) skimmed milk, and then incubated overnight at 4°C in a solution with the primary antibody. A horseradish peroxidase-conjugated secondary antibody was incubated for 1 h at room temperature. Western Lightning ECL reagent Pro (Bio-Rad, CA, U.S.A.) was used to develop the signals, which was captured by ImageQuant™ LAS 4000 biomolecular imager (GE Healthcare Life Sciences, Pennsylvania, U.S.A.) and quantitated by Image J software (National Institute of Health, Bethesda, MD, U.S.A.).

### Enzyme-linked immunosorbent assay (ELISA)

On the 42nd day after AlCl_3_ exposure, six rats were killed and fresh hippocampus tissues were homogenized by T-Per extraction reagent (Pierce Biotechnology, Inc.) to get lysates. After centrifuge, the supernatants were obtained. BDNF ELISA kit (eBioscience, San Diego, CA, U.S.A.) was applied according to the manufacturer’s protocol. A microplate reader (SpectraMax M5, Molecular Devices) at a wavelength of 450 nm was used to detect all standards and samples.

### The ChIP assay

The simple ChIP Enzymatic Chromatin IP Kit (Cell Signaling Technology) was used following the manufacturer’s instruction. In brief, primary antibodies (histone deacetylase 4 [HDAC4], HDAC6, or IgG antibody as a control) were coated to get immunoprecipitated DNA, which was subjected to real-time PCR (RT-PCR) using specific BDNF promoters primers (PI, PII, PIII, PIV, and PIX) (Supplementary Table S1). Then the cumulative fluorescence for each amplification was normalized to the input DNA.

### Real-time polymerase chain reaction (RT-PCR)

The RT-PCR reaction was performed using an ABI STEP ONE RT-PCR System (Applied Biosystems) in a 15-µl volume mixture using 2× FastStart Universal SYBR Green Master Mix (Bio-Rad, Hercules, CA, U.S.A.), which contained 0.375 μM primer and 0.5 μl cDNA. The protocol was followed as: 95°C denaturation for 3 min, 40 cycles of 95°C for 30 s, 60°C for 30 s, finally 72°C for 1 min. Relative gene expression was quantified using the comparative ΔC_T_ method. The relative expression was normalized to GAPDH mRNA expression. Primer sequences were listed in Table 1.

**Table 1:** Primer sequences used for RT-PCR

Gene	Forward Primer (5′-3′)	Reverse Primer (5′-3′)
*Hdac1*	AGTCTGTTACTACTACGACGGG	TGAGCAGCAAATTGTGAGTCAT
*HDAC2*	AGAAGACCCGGACAAAAGAATTT	ACATTCCTACGACCTCCTTCAC
*Hdac3*	GGTTCAGGAAGCCTTCTACCT	ACTCTCTGCTCCAACTTCATACA
*HDAC4*	CCAAGGTTCACCACAGGTCT	TTGTGCCGTAGAGGAGTGTG
*HDAC5*	AGCCCACCACGCTTCTTTG	GTCATCACGGCTGTCATAGGG
*HDAC6*	CAGCACACTTCTTTCCACCAC	AAGTAGGCAGAACCCCCAGT
*HDAC7*	CCTACAGAACTCTTGAGCCCT	GGGGCACTCTCCTTCCTGA
*HDAC8*	ACTATTGCCGGAGATCCAATGT	CCTCCTAAAATCAGAGTTGCCAG
*HDAC9*	GCGGTCCAGGTTAAAACAGAA	GCCACCTCAAACACTCGCTT
*HDAC10*	CAGAGGAAGAGTTGGGCTTG	CATTGTGCACAGCTCCTGTT
*HDAC11*	CAATGGGCATGAGCGAGAC	TGTGGCGGTTGTAGACATCC
*Bdnf P1*	GTGCCTCTCGCCTAGTCATC	CACCATGACTAAGGGTCTCCA
*BdnfP2*	TGAGGATAGGGGTGGAGTTG	GCAGCAGGAGGAAAAGGTTA
*BdnfP3*	GTGTGGTGTGTGTGCGTGTA	GCTAACCTCCTTCCCTCTCC
*BdnfP4*	GCCTGCCCTAGCCTTTACTT	GGCCGGTTTTCTTTTCTTTT
*BdnfP5*	CCCTCCCCCTTTTAACTGAA	GGGGGTGAGGAAAGAGAAAG
*BdnfP6*	CTCCTACTCTGGGGCACATT	ACCGGCTTCTGTCCATTTC
*BdnfP7*	CACGACCCCGAGAGACAG	TCTCTCTCACCACCCTTCCT
*BdnfP8*	TGAGTTTTCATGTGCCCTCT	CAGTCTATATGGCTGGTCAGGA
*BdnfP9*	GAATGCCTTTCCTTGAGGACT	AGCACATCTCTAGGTTTTACTGCAT
*Gapdh*	CTACCCACGGCAAGTTCAAC	CCAGTAGACTCCACGACATA C

### Malondialdehyde (MDA) assay

The thiobarbituric acid (TBA) reaction in the hippocampus was used to determine the level of malondialdehyde (MDA). Briefly, the tissue homogenate was incubated with 10% TCA and 0.67% TBA, and heated at 100°C for 30 min. Then the supernatant was added into a 96-well plate and measured using 532 nm microplate reader (BioTek Instruments, Winooski, VT, U.S.A.). The values were defined as nmol/mg of proteins involved in tissue.

### Superoxide dismutase (SOD) assay

A SOD assay kit (Cayman Company, MI, U.S.A.) was utilized according to the instruction. Briefly, the rat hippocampus was gently homogenized after the 24 h reperfusion. Hypoxanthine and xanthine oxidase were used as the superoxide generator, and nitro blue tetrazolium (NBT) was used as the superoxide indicator, which was measured using a 96-well plate reader (BioTek Instruments, Winooski, VT, U.S.A.) at 450 nm and the SOD activity was defined as U/mg protein.

### Glutathione (GSH) activity

A Glutathione (GSH) assay kit (Cayman Chemical Company, Ann Arbor, MI) was applied to evaluate GSH activity. The homogenized hippocampus tissue was centrifuged (10000 × ***g*** 15 min, 4°C), and 0.6 mmol/l 5,5′-dithiobis(2-nitrobenzoic acid) (DTNB), 0.2 mg/ml NADPH, and glutathione reductase were mixed together to initiate the reaction.

### Glutathione peroxidase (GSH-Px) activity

A glutathione peroxidase (GSH-Px) assay kit (Cayman Chemical Company, Ann Arbor, MI) was applied to evaluate the GSH-Px activity. Briefly, the homogenized hippocampus tissues were centrifuged (10000 × ***g***, 15 min, 4°C). Then 20 μl samples, 100 μl PBS (pH 7.4), 50 μl co-substrate mixture, and 20 μl cumene hydroperoxide were mixed into the 96-well. Finally, a microplate reader (DTX800, Beckman Coulter, Austria) at 340 nm was applied to get the absorbance every minute.

### Statistical analysis

All the data were shown as mean ± SEM. Statistical analysis was performed using one-way ANOVA. Two-way analysis of variance (ANOVA) followed by Tukey’s test for multiple comparisons was applied to analyze the behavioral assessment data. *P*-value ≤0.05 was set as statistical significance.

## Results

### LIPUS recovers aluminium-induced memory acquisition and retrieval attenuation

**Figure 1:**
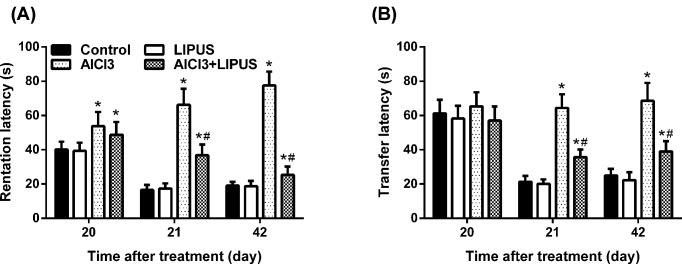
Behavioral effects of LIPUS on AlCl_3_ exposure rats (**A**) Memory retention effect of LIPUS. The AL on the 20th day and RL on the 21st and 42nd day in the AlCl_3_-administrated rats or LIPUS-stimulated group was evaluated. (**B**) Memory performance evaluation of LIPUS. The TL on the 20th, 21st, and 42nd day in AlCl_3_-administrated rats or LIPUS-stimulated group was evaluated. (Results were shown as mean ± s.d., *n*=10 per group; *P<0.05 vs control group, ^#^*P*<0.05 vs AlCl_3_ group).

Consistent with previous reports [10], Morris water maze task results indicated that rats treated with AlCl_3_ had learning and memory deficits when compared with normal control group rats (Figure 1A,B). On day 20, AL of the AlCl_3_-treated group was increased when compared with the control group (*P*<0.05), while AlCl_3_ plus LIPUS could decrease the latency (*P*<0.05). After training, RL was decreased significantly in the normal control group on the 21st and 42nd day when compared with the AlCl_3_ administration group on the 20th day. AlCl_3_ plus LIPUS resulted in a significant decrease in RL on 21st and 42nd day when compared with AlCl3 administration group (*P*<0.05). Moreover, LIPUS plus AlCl3 could decrease RL on the 42nd day when compared with the AlCl3 administration group on the 20th day. All of these results indicated that LIPUS stimulation could improve the latency induced by AlCl3 exposure.

TL referred to the evaluated memory in the elevated plus maze. On the 20th day, there was no significant different mean TL between different groups (Figure 1B). After acclimatization training, there was a significant decrease in mean TL in the normal control and LIPUS groups on the 21st and 42nd day when compared with TL on the 20th day. Compared with pre-trained TL on the 20th day, the mean TL in AlCl_3_-treated group showed no significant differences on the 21st and 42nd day. While, when compared with the normal control group on the 21st and 42nd day a significant increase in TL was observed. Moreover, compared with the TL in AlCl_3_ group, AlCl_3_ plus LIPUS group resulted in a significant decline in TL on the 21st and 42nd day. All of these data suggested that chronic AlCl_3_ exposure markedly deteriorated spatial memory retention and LIPUS stimulation could relieve AlCl_3_-induced deficits in memory acquisition and retrieval.

### LIPUS alleviates the acetylation level of histone H3 lysine 9 and histone H4 lysine 12 (H4K12) reduced by AlCl_3_ exposure

**Figure 2:**
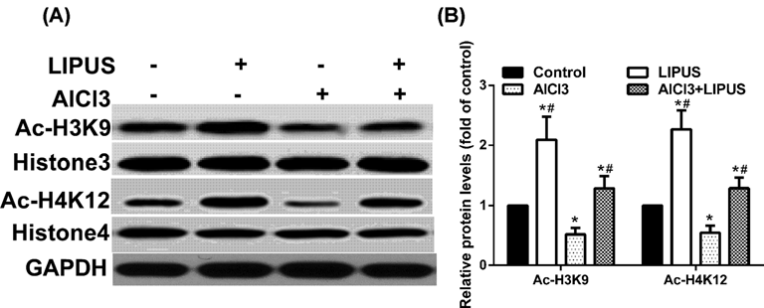
Effects of LIPUS and AlCl_3_ treatment on rat hippocampus histone acetylation The histone acetylation levels of Ac-H3K9 (**A**) and Ac-H4K12 (**B**) were detected by Western blot analysis in the hippocampus. Results were shown as mean ± s.d. (*n*=6). ^*^P < 0.05 vs control group, ^#^*P* < 0.05 vs AlCl_3_ group.

Histone H3 lysine 9 (H3K9) and histone H4 lysine 12 (H4K12) have been correlated with transcriptional activation and chromatin reassembly during DNA replication. As the main brain structure for cognitive function, the hippocampus was utilized to perform further biochemical detection. To investigate whether LIPUS and AlCl_3_ could regulate the acetylation of histones in the hippocampus, the amount of acetyl-H3K9 (Ac-H3K9) and acetyl-H4K12 (Ac-H4K12) were detected (Figure 2A,B). After 42 days of exposure, AlCl_3_ exposure could decrease the acetylation level of H3K9 and H4K12, while LIPUS could alleviate the acetylation level of H3K9 and H4K12 reduced by AlCl_3_ exposure.

### LIPUS reduces the increased expression of HDAC6 exposed to AlCl3

**Figure 3:**
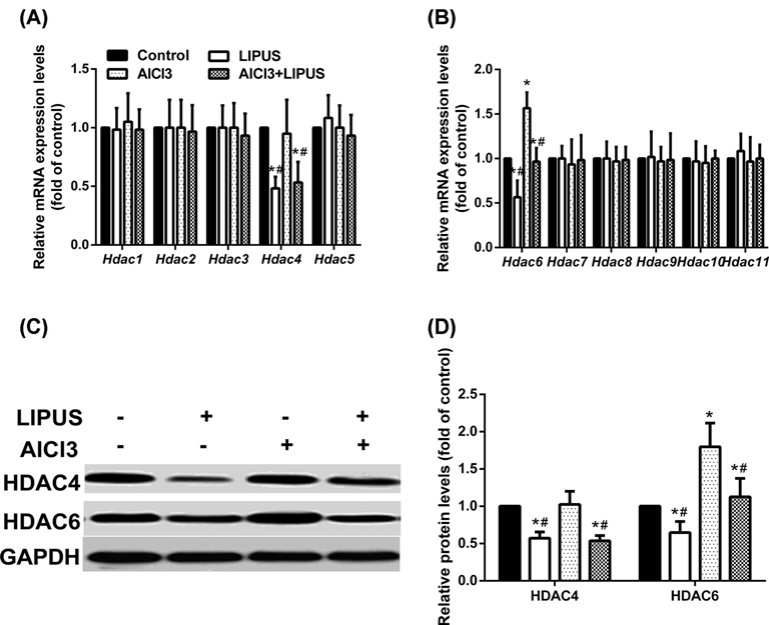
Effects of LIPUS and AlCl_3_ treatment on the expression of HDAC in the rat hippocampus HDAC mRNA levels were measured by qPCR (**A**,**B**), HDAC4 and HDAC6 protein levels were measured by Western blot analyses (**C**,**D**). Results were shown as mean ± s.d. (n = 6). ^*^P < 0.05 vs control group, ^#^*P* < 0.05 vs AlCl_3_ group.

Histone deacetylases (HDAC) can be classified into five subfamilies as Class I (HDAC1, 2, 3, and 8), Class IIa (HDAC4, 5, 7, and 9), Class IIb (HDAC6 and 10), Class III (SIRT1-7), and Class IV (HDAC11). RT-PCR was utilized to determine whether the effects of LIPUS and AlCl3 treatment on H3K9 and H4K12 acetylation were related to HDAC. Only HDAC6 showed differential expression in mRNA level (Figure 3A,B). AlCl_3_ exposure could increase the expression of HDAC6, while LIPUS reduced the marked increase in HDAC6 exposed to AlCl_3_, which suggested the involvement of acetylation in learning and memory modulation.

### LIPUS up-regulates H3K9 and H4K19 acetylation to increase BDNF expression

**Figure 4:**
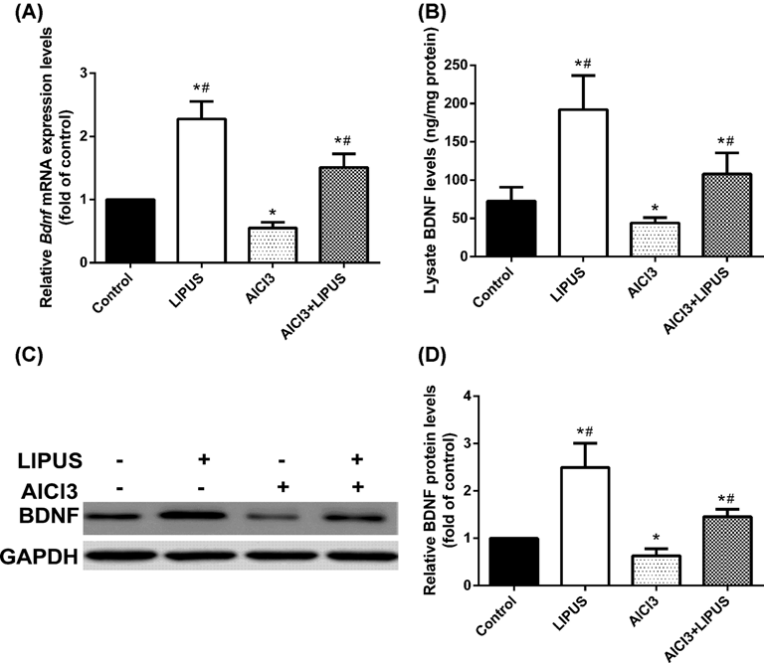
Treatment effects of LIPUS and AlCl_3_ on BDNF mRNA and protein expression in rat hippocampus *Bdnf* mRNA levels were measured by qPCR (**A**). BDNF level in the rat hippocampus lysate was detected by ELISA (**B**). The BDNF protein was detected by Western blot (**C**,**D**). Results were shown as mean ± s.d. (*n*=6). ^*^P < 0.05 vs control group, ^#^*P* < 0.05 vs AlCl3 group.

Previous studies have shown neuroprotection effects associated with BDNF in memory-related disorders. To clarify the link associated with HDACs and BDNF, the mRNA (Figure 4A) and protein (Figure 4B–D) expression of BDNF were measured, and the results showed that LIPUS increased BDNF expression and AlCl_3_ decreased BDNF expression, while LIPUS could regain the reduced expression of BDNF exposed to AlCl3 (Figure 4A–D).

**Figure 5:**
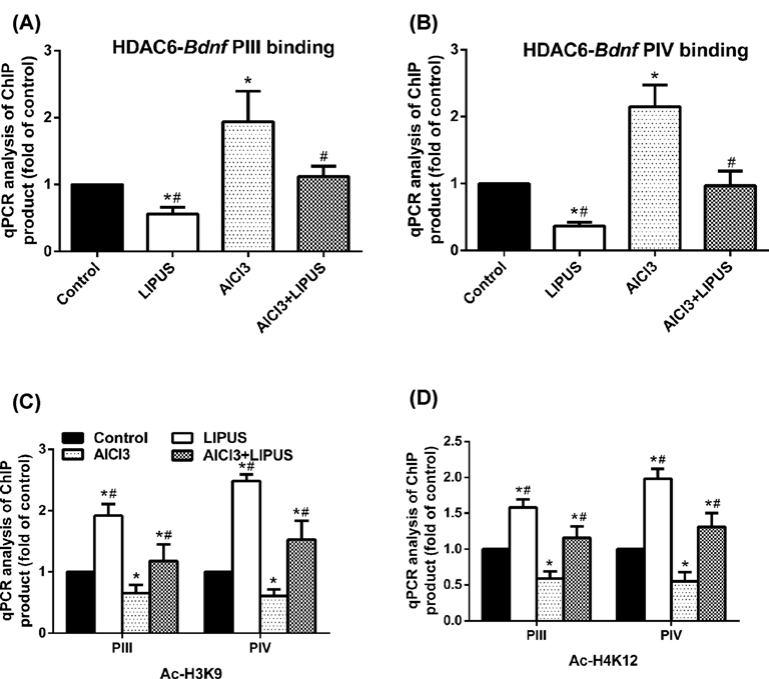
Effects of LIPUS and AlCl_3_ treatment on the HDAC6-PIII and HDAC6-PIV association and the acetylation levels of H3K9 and H4K12 at *bdnf* promoters in rat hippocampus HDAC6-PIII (**A**) and HDAC6-PIV (**B**) association were detected by ChIP and qPCR analyses. The H3K9 (**C**) and H4K12 (**D**) acetylation levels at *bdnf* promoters were detected by ChIP and qPCR. Results were shown as mean ± s.d. (*n*=6). ^*^P < 0.05 vs control group, ^#^*P* < 0.05 vs AlCl_3_ group.

Next, the role of HDAC6 and HDAC4 *BDNF* promoter association were investigated. Chromosome immunoprecipitation analysis showed that there was no association between HDAC6 and *BDNF* PI, PII or PIX (data not shown). LIPUS reduced HDAC6-*BDNF* PIII and PIV association and AlCl_3_ increased it compared with the control group, while LIPUS could block AlCl_3_-induced increase in HDAC6-*BDNF* PIII and PIV association (Figure 5A,B). Coimmunoprecipitated HDAC4 with *BDNF* PIII and PIV showed no significant difference among different groups (data not shown). Histone acetylation has been shown to regulate BDNF expression in various scenarios, we, therefore, measured histone acetylation in the promoter region of BDNF in these rats. LIPUS could cause the increased histone acetylation of H3K9 and H4K19 at *BDNF* PIII and PIV, and AlCl_3_ could lead to reduced histone acetylation of H3K9 and H4K19 at *BDNF* PIII and PIV, while LIPUS could alleviate the reduced histone acetylation of H3K9 and H4K19 at *BDNF* PIII and PIV exposed to AlCl_3_ (Figure 5C,D).

### Effects of LIPUS and AlCl3 treatment on the antioxidant defenses

**Figure 6:**
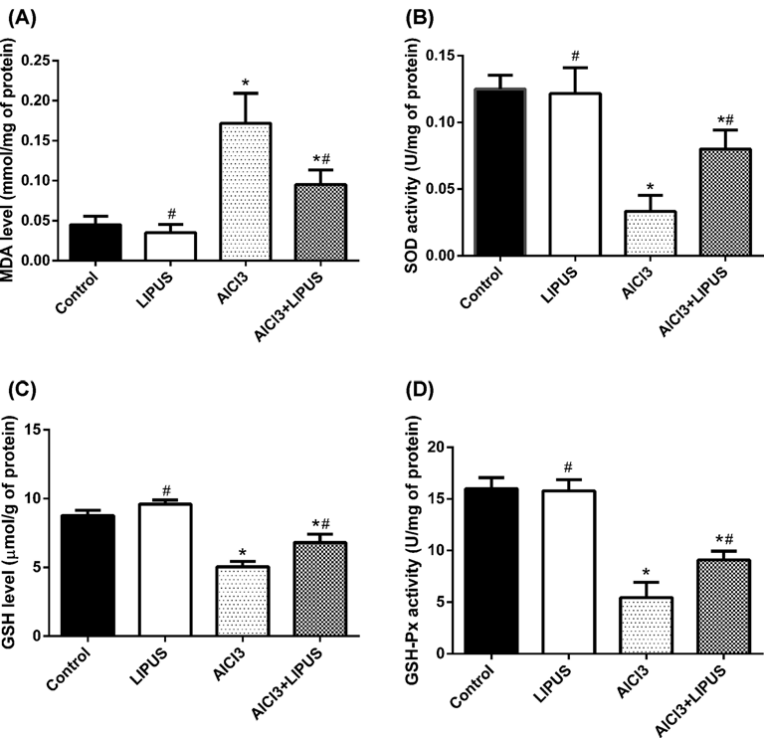
Effects of LIPUS and AlCl_3_ treatment on the antioxidant defenses in rat hippocampus (**A**) The effects of LIPUS and AlCl_3_ on the MDA level in rat hippocampus. (**B**) The effect of LIPUS and AlCl_3_ on SOD activity in rat hippocampus. (**C**) The effect of LIPUS and AlCl_3_ on GSH in rat hippocampus. (**D**) The effect of LIPUS and AlCl_3_ on GSH-Px in rat hippocampus. Results were shown as mean ± s.d. (n = 6). ^*^P < 0.05 vs control group, ^#^*P* < 0.05 vs AlCl_3_ group.

The lipid peroxidation after AlCl_3_ exposure was demonstrated by MDA level and the neuroprotective effect of antioxidant enzyme activities. The data suggested a significant MDA elevation in AlCl_3_ exposure group compared with LIPUS and control group in the hippocampus (Figure 6A). AlCl_3_ exposure significantly decreased the activities of SOD, GSH, and GSH-Px (Figure 6B–D, respectively), while LIPUS can recover the antioxidant enzyme activities decreased by AlCl_3_.

## Discussion

Neurodegenerative diseases-associated chronic cerebral damages seriously affect the quality and length of human life. Nevertheless, the etiology and pathogenesis mechanism involved in cerebral damage is not clearly illustrated. So, it is vital to further define the mechanisms involved in cerebral damage and put forward effective treatment strategy. The current study shows that AlCl_3_ exposure leads to impaired spatial learning and memory, which is accompanied by decreased H3K9 and H4K19 acetylation, reduced BDNF expression and increased oxidative stress. While LIPUS treatment is sufficient to alleviate reduced histone acetylation and cognitive dysfunction in AlCl3 exposed rat.

Aluminium salts are considered as potent neurotoxins, which may pose many adverse effects on the central nervous system *in vitro* and induce neurofibrillary degeneration in animal models [11]. While, in biological tissue or *in vivo*, aluminium salts just have the potential to promote reactive oxygen species (ROS) formation and do not show any direct pro-oxidant properties [12]. It is worth noting that the connection between Alzheimer’s disease and aluminium salts is still in controversy. Experimentally, chronic exposure to aluminium can cause neuropathological changes and cognitive impairments, which are similar to those of Alzheimer’s disease, including the deficiency of the neurotransmitter acetylcholine, extracellular amyloid β (Aβ) deposits, neurofibrillary tangles, and the loss of neurons [13,14]. So, the relevant research in aluminium exposure can give vital application for the prevention and therapy of Alzheimer’s disease.

It is reported that HDACs-relevant histone deacetylation is involved in the regulation of memory acquisition and maintenance on both physiological and pathological conditions [15]. Dysregulated histone acetylation is observed in old mice with age-induced memory impairment and restoration of histone acetylation can recover cognitive function and memory-associated gene expression [16,17]. In aged rats, it is reported that sodium butyrate (histone deacetylase inhibitor) not only reverses the signaling changes including down-regulation of HDAC2 and restoration of BDNF expression but also rescues cognitive impairment caused by repeated isoflurane exposure [18]. Similarly, repeated isoflurane exposure to neonatal mice also results in cognitive deficit and abnormal histone acetylation in the hippocampus, a brain region which is important for learning and memory in adulthood [19]. Therapies confronting histone acetylation regulation in the hippocampus may be future new directions.

As an important neurotrophic factor involved in various neuronal functions, BDNF levels positively correlate with cognitive function. For instance, the insufficient level of BDNF has a vital role in the development and pathology of Alzheimer’s disease, Parkinson’s disease, and Huntington’s disease [20], while recombinant BDNF improves cognition and increases hippocampal synapse density in mice model of Alzheimer’s disease [21,22]. In general, LIPUS may offer a new solution to the endogenous enhancement of BDNF through neural circuits stimulation to circumvent the blood–brain barrier.

It is testified that LIPUS can promote bone and tissue regeneration and have a positive improvement on axonal regeneration showed by *in vivo* peripheral nerve injury trials [23]. The locally applied ultrasound stimuli could markedly increase the number of Schwann cell nuclei and the expression of BDNF on the damaged sciatic nerve [24]. LIPUS reverses the results of aluminium on BDNF expression and oxidative stress. We believe such regulation is through histone acetylation since we found that acetylation of H3K9 and H4K12 at the PIII and PIV promoter of BDNF is suppressed by aluminium exposure and alleviated by LIPUS treatment. Given that decreased histone acetylation and BDNF levels are associated with aging-related cognitive decline and that patients with Alzheimer’s disease have reduced BDNF expression [25], LIPUS may also be beneficial to cognitive function in aging and Alzheimer’s disease.

It is worth mentioning that LIPUS alone cannot enhance the anti-oxidative enzymes activity, while it can recover the antioxidant enzyme activities decreased by AlCl_3_ and decrease the oxidative stress in the AlCl_3_-treated hippocampus. AlCl_3_ may cause excitatory damage to neurons and LIPUS alone or combined with AlCl_3_ could up-regulate the expression of BDNF when compared with control group, such mechanisms may contribute to the decrease in oxidative stress.

In our study, we demonstrate that LIPUS is sufficient to alleviate histone acetylation and cognitive dysfunction in aluminum exposed rats through acetylation regulation of BDNF. Whether LIPUS can be utilized to perform some pre-clinical trials, may need to be testified in more models.

## Abbreviations

AL: acquisition latency
AlCl_3_: aluminum chloride
BDNF: brain-derived neurotrophic factor
GSH: glutathione
GSH-Px: glutathione peroxidase
H3K9: histone H3 lysine 9
H4K12: histone H4 lysine 12
HDAC: histone deacetylase
LIPUS: low intensity pulsed ultrasound
MDA: malondialdehyde
RL: retention latency
SDD: Sprague–Dawley
SOD: superoxide dismutase
TL: transfer latency
TBA: thiobarbituric acid
